# Preparation of an Aminated Lignin/Fe(III)/Polyvinyl Alcohol Film: A Packaging Material with UV Resistance and Slow-Release Function

**DOI:** 10.3390/foods12142794

**Published:** 2023-07-23

**Authors:** Shushan Gao, Chonghao Zhu, Liangfei Ma, Chenghai Liu, Hongqiong Zhang, Shengming Zhang

**Affiliations:** 1 College of Engineering, Northeast Agricultural University, Harbin 150030, China; a07190058@neau.edu.cn (S.G.); s210702023@neau.edu.cn (C.Z.); s220701029@neau.edu.cn (L.M.); liuchenghai@neau.edu.cn (C.L.); zhhqiong@neau.edu.cn (H.Z.); 2 Key Laboratory of Pig-Breeding Facilities Engineering, Ministry of Agriculture and Rural Affairs, Harbin 150030, China; 3 Heilongjiang Province Technology Innovation Center of Mechanization and Materialization of Major Crops Production, Harbin 150030, China

**Keywords:** lignin, polyvinyl alcohol, Fe(III), packaging material, slow-release function

## Abstract

To reduce the usage of petroleum-based plastic products, a lignin-based film material named aminated lignin/Fe(III)/PVA was developed. The mixture of 8 g lignin, 12 mL diethylenetriamine, 200 mL NaOH solution (0.4 mol·L^−1^), and 8 mL formaldehyde was heated at 85 °C for 4 h; after the aminated lignin was impregnated in the Fe(NO_3_)_3_ solution, a mixture of 3 g aminated lignin/Fe(III), 7 g PVA, and 200 mL NaOH solution (pH 8) was heated at 85 °C for 60 min; after 2 mL of glycerin was added, the mixture was spread on a glass plate to obtain the aminated lignin/Fe(III)/PVA film. This film demonstrated hydrophobicity, an UV-blocking function, and a good slow-release performance. Due to the formation of hydrogen bonds between the hydroxyl groups of lignin and PVA, the tensile strength, the elongation at break, and the fracture resistance of the film were 9.1%, 107.8%, and 21.9% higher than that of pure PVA film, respectively. The iron content of aminated lignin/Fe(III)/PVA was 1.06 wt%, which mainly existed in a trivalent form. The aminated lignin/Fe(III)/PVA film has the potential to be used as a food packaging material with anti-ultraviolet light function and can also be developed as other packaging materials, such as seedling bowls, pots for transplanting, and coating films during transport.

## 1. Introduction

Lignin is the second most abundant organic matter in plants. It possesses the advantages of being an abundant source, and is non-toxic, biodegradable, and inexpensive [1,2]. Lignin is mainly composed of phenylpropane structural units connected by carbon–carbon bonds and ether bonds [3]. The rich functional groups along with the large number of chemical reaction active sites present in lignin are conducive to further expanding the functions of lignin through chemical modification approaches, such as hydrogen alkylation, amination, nitration, sulfation, sulfonation, alkylation/dealkylation, esterification, and pharmacology [4,5]. In recent years, lignin, as a biomass material, along with its inherent biodegradability and biocompatibility, have attracted widespread levels of attention and research in the fields of green sustainable agriculture and new packaging materials [6,7].

Current commercially available seedling bowls and plant transplanting pots are still dominated by petroleum-based materials, such as plastics, which are difficult to biodegrade and are environmentally unfriendly [8,9]. The development and preparation of lignin-based films or flake packaging materials will effectively solve the above problems. However, pure lignin does not have film-forming properties, meaning it usually needs to be processed with some other polymer materials to improve the mechanical properties of the lignin-based materials [10]. Polyvinyl alcohol (PVA) is a highly polar and water-soluble polymer, which has been widely used in the preparation of green polymer materials due to its advantages, which include a film-forming ability, biodegradability, non-toxicity, and biocompatibility [11]. Unlike other petroleum-based plastics, PVA is a rare, degradable polymer. But its natural degradation rate is relatively slow [12]. Korbag et al. [13] found that adding an appropriate amount of lignin filler into the blend material matrix could improve the degradation rate of PVA. Moreover, the strong intermolecular interactions between the lignin molecules and the PVA hydroxyl groups increased the mechanical strength of these blends. In previous studies, we found that the addition of the trimethyl lignin quaternary ammonium salt improved the stability of the self-assembled TLQA/CMPVA films in water [14]. In addition, a few PVA-based functional films have also been developed. Wang et al. [15] synthesized soy protein isolate/oxidized cross/sign films using chemical/physical interactions, which were able to improve the germination rate of cabbage seeds. Tian et al. [16] produced a lignin-doped PVA liquid mulch using a cyanoethylation technological route, which improved the barrier property of the raw film materials and slowed down the photodegradation rate of photosensitive pesticides.

Iron (Fe) is an essential nutrient element involved in the synthesis of plant chloroplasts [17]. Supplementing transplanted seedlings with iron is beneficial for increasing the photosynthetic rate and promoting plant growth. Previous studies have shown that an excessive supplementation of iron can lead to the manifestation of various metabolic disorders and even cause the death of the plants [18]. Therefore, divalent ferric (Fe(II)) liquid fertilizer is usually used as a foliar fertilizer, which is applied by spraying the plant leaves multiple times [19]. Trivalent iron(Fe(III)) solid fertilizer is commonly used as a base fertilizer, and it is expected that this fertilizer has a long-term and slow-release function [20]. Nowadays, there has been a rapid development in the preparation technology and mechanism research of the lignin-based, slow-release fertilizers. Jiao et al. [21] extended the release cycle of nitrogen by complexing amino groups onto hydroxylated lignin macromolecules through the Mannich reaction. Li et al. [22] prepared a lignin-based magnetic fertilizer (M/ALFeF) based on the chelation reaction and electrostatic adsorption principles. The nutrient release of this fertilizer was found to be consistent with the fertilizer requirements for crop growth.

Based on the above points, we attempted to prepare a new lignin-based packaging material with iron slow-release properties termed aminated lignin/Fe(III)/PVA (AL/Fe(III)/PVA). The aminated lignin was synthesized through the Mannich reaction pathway. The iron element was combined with aminated lignin through the complexation method to produce aminated lignin/Fe(III) (AL/Fe(III)) particles. The particles were blended with polyvinyl alcohol, and then the film material was prepared using the flow casting method. The AL/Fe(III)/PVA material has the following potential applications. On the one hand, it can be processed into films, which can be used as food packaging, seedling bowls, and plant transplanting pots. On the other hand, the materials in a molten state can be used for root treatment in seedling transportation packaging. The appropriate coating techniques were used to cover and fix the soil close to the seedling roots, thereby shortening the slow seedling period.

## 2. Materials and Methods

### 2.1. Materials

The lignin (dealkaline), polyvinyl alcohol (1795), diethylenetriamine (DETA), and iron nitrate nonahydrate were all purchased from Aladdin Biochemical Technology Co., Ltd. (Shanghai, China). The anhydrous ethanol, ethylene glycol, formaldehyde, hydrochloric acid (HCl), sodium hydroxide (NaOH), and glycerol were purchased from Tianjin Chemical Reagent Factory (Tianjin, China). All the reagents used were of analytical grade except for lignin and polyvinyl alcohol.

### 2.2. Preparation Method of the AL/Fe(III)/PVA Film

The preparation method of aminated lignin was referred to the study published by the authors of [23]. Briefly, a mixture of 8.0 g lignin, 200 mL NaOH solution (0.4 mol·L^−1^), and 12.0 mL DETA was heated at 85 °C with magnetic stirring. Then, 8.0 mL of formaldehyde solution was added dropwise into the mixture and then was continued to be heated at 85 °C and stirred for 4 h. The mixture was adjusted to pH 4.5 with 1.0 mol·L^−1^ of HCl solution, following which it was vacuum filtered. The solid residue was washed to neutral with distilled water and vacuum dried at 55 °C for 24 h with a vacuum of 0.08 MPa to obtain the aminated lignin. Following this, 5 mL of Fe(NO_3_)_3_ solution was added dropwise into a mixture of 4.0 g aminated lignin and 200 mL deionized water. After the mixture was magnetically stirred at room temperature for 6 h, it was centrifuged at 4000 rpm for 5 min. Then, 50 mL of deionized water was mixed with the precipitate and centrifuged again. This step was performed three times in total. Finally, the precipitate was vacuum dried at 55 °C for 24 h to obtain the AL/Fe(III). The effect of the Fe(NO_3_)_3_ solution weight concentration was investigated at 3%, 4%, 5%, 6%, and 7%, respectively.

PVA and 200 mL of solution (adjusting with 0.1 mol·L^−1^ hydrochloric acid or sodium hydroxide solution and distilled water) were added to a round-bottomed flask. The mixture was then magnetically stirred and heated for 1 h. The effects of the pH value (at 4, 6, 8, 9, and 11, respectively) of the solution and the reaction temperature (at 65, 75, 85, 95, and 105 °C, respectively) were assessed. The AL/Fe(III) was added into the mixture, and the mixture was then continuously heated with magnetic stirring at 300 rpm. The total addition mass of AL/Fe(III) and PVA was 10 g. The effect of the mass ratio of the two materials was assessed at the ratios of 0:10, 1:9, 1:4, 3:7, 2:3, and 1:1, respectively. The effect of the reaction time was examined at 30, 45, 60, 75, and 90 min, respectively. After that, 2 mL of glycerin was poured into the mixture and stirred for another 30 min. Finally, about 10 mL of the mixture was spread on a glass plate and dried at room temperature for 24 h to obtain the AL/Fe(III)/PVA film.

### 2.3. Performance Evaluation of the AL/Fe(III)/PVA Film

#### 2.3.1. Mechanical Performance

The tensile performance of the different film samples was conducted using an IMT-202A universal material tensile testing machine (Dongguan Yingte Naisen Precision Instrument Co., Ltd., Dongguan, China). The film was cut into strips of 100 mm × 10 mm and then clamped on the test machine. The loading speed was set at a constant speed of 50 mm per minute. The tensile strength and elongation at break were calculated according to the following equations:

(1) $$S=\frac{F}{b},$$

(2) $$\varepsilon =\frac{∆l}{l}.$$

where *S* refers to the tensile strength, kN·m^−1^; *F* refers to the maximum tensile resistance, N; *b* refers to the width of the sample, mm; *ε* refers to the elongation at break; ∆*l* refers to the elongation at fracture, mm; and *l* refers to the initial length of the sample, mm.

The breaking strength was determined using a YT-NPY5600Q cardboard rupture tester (Hangzhou Yante Technology Co., Ltd., Hangzhou, China). Prior to testing, the film was cut into a 70 mm × 70 mm square and fixed on the testing machine.

#### 2.3.2. UV Light-Shielding Performance

The film was cut into strips of 40 mm × 10 mm, following which it was assessed using a UV-2600 dual-beam UV–visible spectrophotometer (Shanghai Leiyun Test Instrument Manufacturing Co., Ltd., Shanghai, China). The scan wavelength was in a range of 200–800 nm and the resolution was 1 nm. Moreover, the UV light at wavelength 365 nm was used to further evaluate the resistance of the film to UV light. The ultraviolet lamp was used to shine the 100 Yuan RMB to observe for the presence of any anti-counterfeiting signs.

#### 2.3.3. Slow-Release Performance Analysis

The soil column leaching test was used to detect the release behavior of the iron in the AL/Fe(III)/PVA film. The test was performed in a polymethyl methacrylate tube with an inner diameter of 4.72 cm and a height of 15 cm. The method was referred to the methodology outlined by the authors of [24] with some modifications. The film was cut into squares of 10 mm × 10 mm. Approximately 0.2 g of film fragments and 50 g of quartz sand were filled into the tube, in which the filling depth of the film fragments was about 1 cm from the surface. An initial volume of 220 mL of distilled water was added to the tube, and 25 mL of distilled water was added to the tube on the 1st, 3rd, 5th, 7th, 10th, 15th, 20th, 25th, and 30th day, respectively. After each addition of distilled water, the valve at the bottom of the tube was opened and 25 mL of solution was taken out to measure the iron content using an atomic absorption spectrophotometer (iCE3500, Thermo Fisher Scientific Inc., Waltham, MA, USA). Different kinetic models were used to fit the slow-release data of the nutrient element iron, and their slow-release pattern was analyzed. The correlation coefficient R^2^ obtained was used to evaluate its fitting degree. The closer the R^2^ value was to 1, the better the model fitted.

### 2.4. Characterization Method of the AL/Fe(III)/PVA Film

The contact angle of the films was measured with 5 μL of deionized water on an OCA20 video optical contact angle meter (DataPhysics Instruments, Stuttgart, Germany). The morphology of the film was analyzed using an S-3400N tungsten filament scanning electron microscope (Hitachi Corporation, Tokyo, Japan). The magnification was set as 5000 times. The functional groups of the film samples were assessed using an Agilent Cary 630 FT-IR spectrometer (Agilent Technologies Inc., Santa Clara, CA, USA). The scanning range was in a range of 400–4000 cm^−1^ and the resolution was 2 cm^−1^. An Agilent 7800 inductively coupled plasma mass spectrometer (Agilent Technologies Inc., Santa Clara, CA, USA) was used for the determination of iron in film samples. The crystallinity of the samples was measured using a Rigaku Ultima IV X-ray diffractometer (Rigaku Corporation, Saitama, Japan) with Cu Kα radiation. The range of the scanning angles were from 5° to 80°, respectively, and the scanning speed was 4°·min^−1^. XPS of the samples were tested using an AXIS Supra+TM X-ray photoelectron spectrometer (Shimadzu Corporation, Tokyo, Japan) with a monochromatic Al Kα source. The photon energy (hv) was 1486.6 eV.

### 2.5. Statistical Analysis

The tests of the mechanical properties of the film samples were performed in triplicates. The data were analyzed statistically through conducting the analysis of variance (ANOVA) statistical test using the software SPSS 17.0 (IBM (China) Investment Co., Ltd., Shanghai, China), and were presented as mean ± standard deviation.

## 3. Results and Discussion

### 3.1. Effect of Preparation Conditions on the Properties of the Films

**Figure 1:**
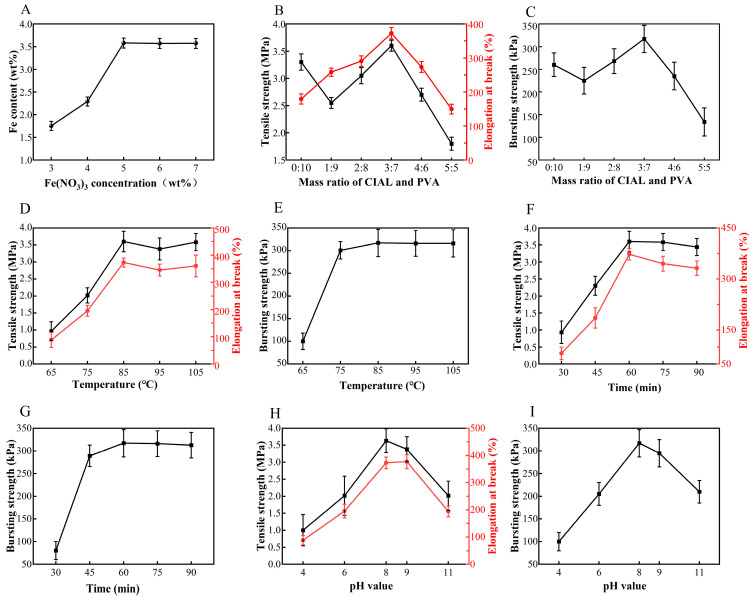
The effect of the concentration of ferric nitrate solution on the iron content of AL/Fe(III) (**A**). The effect of the mass ratio of AL/Fe(III) to PVA on the tensile strength, elongation at break (**B**), and breakage resistance (**C**) of the film materials. The effect of the reaction temperature on the tensile strength, elongation at break (**D**), and breakage resistance (**E**) of the film materials. The effect of the reaction time on the tensile strength, elongation at break (**F**), and breakage resistance (**G**) of the film materials. The effect of the pH value on the tensile strength, elongation at break (**H**), and breakage resistance (**I**) of the film materials.

Figure 1A shows the effect of the weight concentration of the ferric nitrate solution on the iron content of AL/Fe(III). When the weight concentration of the Fe(NO_3_)_3_ solution was 3% and 4%, respectively, the iron content of AL/Fe(III) was 1.76 wt% and 2.29 wt%, respectively. When the weight concentration of the Fe(NO_3_)_3_ solution increased to 5%, the iron content of AL/Fe(III) reached 3.58 wt%. However, as the weight concentration of Fe(NO_3_)_3_ solution continued to increase, the iron content of the prepared AL/Fe(III) did not increase, indicating that the maximum loading capacity of the aminated lignin had been reached. Therefore, the optimum Fe(NO_3_)_3_ solution weight concentration for the preparation of AL/Fe(III) was 5%. Under this condition, the iron content of the further prepared AL/Fe(III)/PVA film was 1.06 wt%.

Figure 1B shows the effect of the mass ratio of AL/Fe(III) to PVA on the tensile strength and the elongation at break of the film materials under the reaction conditions of 85 °C, 60 min, and pH 8. As the mass ratio of AL/Fe(III) to PVA increased, the tensile strength and elongation at break of the film initially showed an increasing trend followed by a decreasing trend. When a small amount of AL/Fe(III) was added, such as a mass ratio of 1:9, the tensile strength of the film was 2.55 MPa, indicating a decrease of 29.4% compared to the pure PVA film (3.3 Mpa). This observed phenomenon was deemed to be due to the uneven distribution of a small amount of AL/Fe(III) in the PVA film, which subsequently damaged the intermolecular structure of the pure PVA film. When the mass ratio of AL/Fe(III) to PVA was 3:7, the tensile strength and elongation at break of the film reached a maximum of 3.6 MPa and 372.7%, respectively, which indicated an increase of 9.1% and 107.8% compared to the pure PVA film, respectively. This phenomenon may have been observed due to PVA being a rigid ion, the lignin fraction containing reactive groups (e.g., phenolic hydroxyl and alcohol hydroxyl), along with the co-blending of the lignin hydroxyl group hydrogen bonds formed between the hydroxyl groups of lignin and PVA to enhance the tensile strength of the films [25]. Figure 1C shows the effect of the mass ratio on the breakage resistance of the film materials. With the increase in the mass ratio of AL/Fe(III) to PVA, the breaking resistance of these films first increased and then decreased. When the mass ratio was 1:9, the breakage resistance of the AL/Fe(III)/PVA film was 34.9 kPa lower than that of the pure PVA film (260 kPa), indicating that the mechanical properties of the composite film was worse than that of the pure PVA film. The optimal value of the breakage resistance of the film was 317.0 kPa at a mass ratio of 3:7, which was 21.9% higher than that of the pure PVA film. Therefore, the mechanical properties of this composite film can be improved by adding an appropriate amount of AL/Fe(III). The mass ratio of AL/Fe(III) to PVA was determined to be 3:7.

Figure 1D,E display the effects of the reaction temperature on the tensile strength, elongation at break, and breakage resistance of the film materials under the reaction conditions of 60 min, pH 8, and a mass ratio of 3:7. When the reaction temperature was 65 °C, the values of the above mechanical properties of the composite film were 0.96 MPa, 88.13%, and 101.0 kPa, respectively. With the increase in the reaction temperature, the mechanical properties of the composite films also showed an increasing trend. When the reaction temperature was 85 °C, the above three mechanical properties reached 3.6 MPa, 372.7%, and 317 kPa, respectively. After that, increasing the reaction temperature had no significant effect on the mechanical properties of the composite films. The analysis results showed that when the reaction temperature was lower than 85 °C, the reaction did not occur completely, resulting in poor mechanical properties of the composite film. The optimum reaction temperature was thus determined to be 85 °C.

Figure 1F,G show the effects of the reaction time on the mechanical properties of the films under the reaction conditions of 85 °C, pH 8, and a mass ratio of 3:7. In the time range from 30 to 60 min, respectively, the tensile strength, elongation at break, and fracture resistance of the film materials increased with the increase in the reaction time. When the reaction time was 60 min, the above three mechanical property indexes reached their maximum values of 3.6 MPa, 372.7%, and 317 kPa, respectively. With the increase in the reaction time, the tensile strength and fracture resistance of the film material did not increase, but the elongation at break decreased slightly. This phenomenon was determined to be due to the short reaction time and the crosslinking process between PVA and the chelated iron amine lignin being insufficient. In contrast, having too long of a reaction time might destroy the complete crosslinking process between PVA and AL/Fe(III), resulting in a slight decrease in the mechanical properties of the film. Therefore, the reaction time was set as 60 min.

The effect of the pH value on the mechanical properties of the films is shown in Figure 1H,I. The results showed that the mechanical properties of the composite films were the best at the value of pH 8, especially in terms of the tensile strength. Under acidic conditions, AL/Fe(III) would precipitate to some extent, which would therefore not be conducive to the solution cross-linking reaction, and result in an uneven distribution of the cross-linked film. If the solution was too basic, the cross-linking reaction might be somewhat restricted, and the mechanical properties of the film would be deteriorated as a result.

Zhou et al. [26] found that the mechanical properties of the PVA–chitin nanofiber composite film were improved by adding a 1 wt% of lignin nanoparticles. Li et al. [27] found that when the weight content of alkaline lignin was 5%, the tensile strength and fracture strain of the PVA–alkaline lignin composite film were significantly enhanced. In the process of stretching the composites, lignin function as intermolecular sacrificial hydrogen bonds, limiting the movement of different molecules, thereby improving the mechanical properties of the composites [28]. However, the addition of excessive lignin in the composite film will cause a certain degree of microphase separation, resulting in a decrease in the tensile strain [13]. Even so, the rigid aromatic structure of lignin enhances the mechanical properties of the AL/Fe(III)/PVA film, which is beneficial for the development of these AL/Fe(III)/PVA films as packaging materials.

### 3.2. Characterization of the AL/Fe(III)/PVA Film

**Figure 2:**
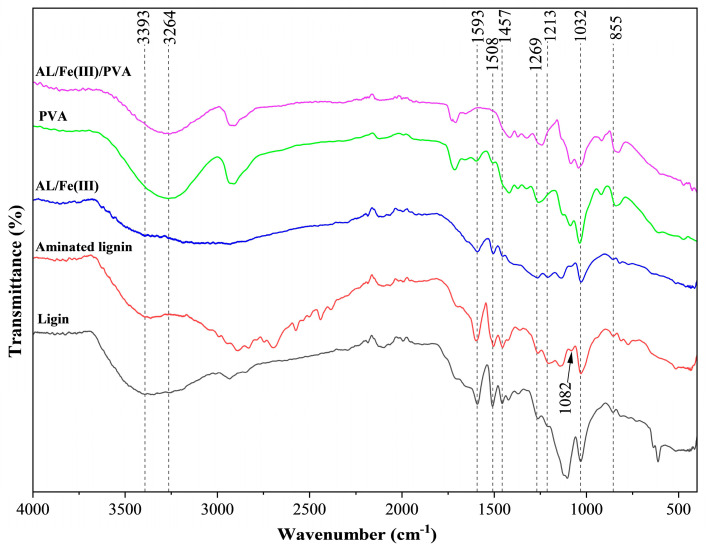
The FT-IR spectra of lignin, aminated lignin, AL/Fe(III), PVA, and AL/Fe(III)/PVA, respectively.

The FT-IR spectra of different materials are displayed in Figure 2. A few typical lignin absorption peaks can be clearly observed from the spectra of lignin, aminated lignin, and AL/Fe(III). The wide peak at 3393 cm^−1^ was assigned to the hydroxyl groups present in the aliphatic and phenolic structures [29]. The peaks observed at 1603 cm^−1^ and 1506 cm^−1^ were attributed to the aromatic skeletal stretching vibrations. The peaks at 1457 cm^−1^ and 855 cm^−1^ were determined to be derived from the C–H single bond bending and out-of-plane deform vibration processes, respectively [30]. In the aromatic structures, the peaks observed at 1269 cm^−1^ and 1213 cm^−1^ were assigned to the guaiacyl and syringyl structures, respectively, and the peak at 1032 cm^−1^ originated from an ether bond in the lignin structure [31,32,33], suggesting that the skeleton structure of lignin was not destroyed during the Mannich reaction. As the Mannich reaction occurred in the aromatic region of lignin, the intensity of the peaks of the C–H single bond vibrations in the aromatic skeleton decreased significantly in the spectra of aminated lignin and AL/Fe(III), such as the peaks observed at 1593 cm^−1^, 1508 cm^−1^, 1457 cm^−1^, and 855 cm^−1^, respectively. In addition, in the spectra of aminated lignin and AL/Fe(III), a new peak at 1082 cm^−1^ was observed, which was attributed to the C–N stretching and bending vibrations [31], implying that the amino groups were grafted into the lignin structure. In the spectra of AL/Fe(III), the intensity of the broad peak observed at 3393 cm^−1^ was significantly weakened due to the attachment of iron [34]. Compared with the spectra of pure PVA, the peak of the typical hydroxyl (O–H) stretching vibration in the spectra of AL/Fe(III)/PVA was blue-shifted from 3264 cm^−1^ to 3270 cm^−1^, respectively. This was determined to be due to the strong hydrogen bonds formed by the presence of a large number of hydroxyl groups between the PVA and AL/Fe(III) complexes [35]. Based on the above results, the addition of the AL/Fe(III) complex did not affect the PVA structure, indicating that the reaction between these two materials was mainly a physically bonded blending reaction rather than a chemical reaction.

**Figure 3:**
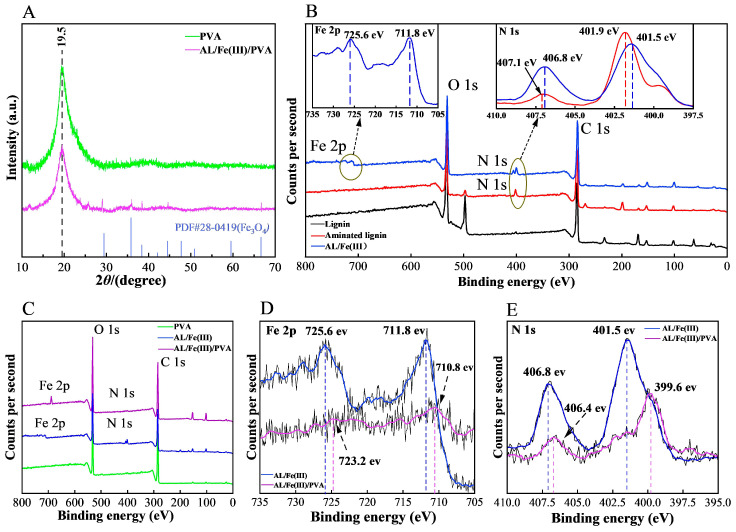
The XRD patterns of PVA and AL/Fe(III)/PVA (**A**). The XPS survey of lignin, aminated lignin, and AL/Fe(III) (**B**). The XPS survey of AL/Fe(III), PVA, and the AL/Fe(III)/PVA film (**C**). Fe 2p scan of AL/Fe(III) and the AL/Fe(III)/PVA film (**D**). N 1s scan of AL/Fe(III) and the AL/Fe(III)/PVA film (**E**).

Figure 3A shows the XRD spectra of PVA and AL/Fe(III)/PVA. In the spectra of PVA, the sharp peak at 19.5° corresponded to the (101) crystalline plane [36], and non-significant broad diffraction peaks were also observed at 11° and 40°, respectively [37]. These findings indicate that PVA is a semi-crystalline substance encompassing crystalline and amorphous regions. Compared with the spectra of PVA, the diffraction peak intensity observed at 19.4° in the spectra of AL/Fe(III)/PVA was reduced. This phenomenon was determined to have been caused by the cross-linking of aminated lignin/Fe(III) with the hydroxyl groups of PVA, which destroys the crystalline region of the PVA molecule. Furthermore, in the spectra of AL/Fe(III)/PVA, a few new peaks were observed at 29°, 36°, 38°, 44°, and 67°, respectively. The above enhanced and emergent peaks match the standard card for Fe_3_O_4_ (PDF#28-0419), indicating that iron was successfully loaded onto the AL/Fe(III)/PVA films.

Figure 3B shows the XPS survey of lignin, aminated lignin, and AL/Fe(III). Compared with the spectra of lignin, the characteristic peak of N 1s was observed in the spectra of aminated lignin and AL/Fe(III), indicating that the lignin was successfully aminated. Moreover, the binding energies for Fe 2p were observed in the spectra of AL/Fe(III), which indicated that the iron was successfully complexed to the aminating lignin. More specifically, the N 1s spectrum of AL displayed signals at 401.9 eV and 407.1 eV, corresponding to the substituted amine and amine group binding energies, respectively [38]. After the aminated lignin was complexed with iron, the binding energy at 401.9 eV was shifted to the lower binding energy at 401.5 eV, which could be attributed to the formation of Fe–N due to the higher electronegativity of iron compared to nitrogen. This result is consistent with the FT-IR analysis, confirming that due to the presence of the amino groups, the aminated lignin had a strong ability to complex the transition metal ions. In addition, the binding energies of 725.6 eV (for Fe 2p_1/2_) and 711.8 eV (for Fe 2p_3/2_) corresponded to Fe^3+^ [34], indicating that the iron element present was mainly trivalent iron.

Figure 3C shows the XPS survey of PVA, AL/Fe(III), and AL/Fe(III)/PVA. Compared with the spectra of PVA, the binding energies for N 1s and Fe 2p were observed in the spectra of the other two materials, which was caused by the AL/Fe(III) in the composite film. In Figure 3D, the binding energies for the Fe 2p of AL/Fe(III)/PVA observed at 725.6 eV and 711.8 eV showed a slight blue-shift. In the spectra of AL/Fe(III)/PVA, the weakened binding energies at 710.8 eV and 723.2 eV may have been caused by the presence of strong hydrogen bonds between AL/Fe(III) and PVA in the film material [23]. Meanwhile, in Figure 3E, compared with the spectra of AL/Fe(III), the binding energies for the N 1s of the AL/Fe(III)/PVA at 399.6 eV and 406.4 eV showed a slight blue-shift.

### 3.3. UV Light-Shielding Properties of the Films

**Figure 4:**
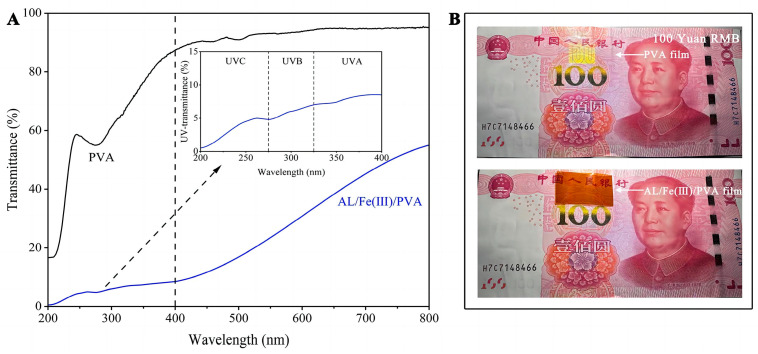
The UV–visible transmittance curves of the PVA and AL/Fe(III)/PVA films (**A**). The photograph of a RMB covered with the PVA film or the AL/Fe(III)/PVA film under ultraviolet light irradiation (**B**).

The UV–visible transmittance curves for the PVA and AL/Fe(III)/PVA films are shown in Figure 4A. The AL/Fe(III)/PVA film can shield almost 100% of the UVB (325–275 nm) and UVC (275–200 nm) and most of the UVA (400–325 nm) spectrum, indicating that the AL/Fe(III)/PVA film exhibits strong UV-shielding properties. Moreover, the shielding effect of the AL/Fe(III)/PVA film on ultraviolet light was evaluated using the method entailing the illumination of the RMB with the ultraviolet light, as shown in Figure 4B. Under ultraviolet light, the RMB will display fluorescent anti-counterfeit markings. The results showed that the PVA film had no shielding effect against UV light, with the anti-counterfeiting mark on 100 Yuan RMB appearing under ultraviolet light. When the AL/Fe(III) was added to the PVA, the composite film had a strong ultraviolet shielding performance, and no anti-counterfeiting mark was observed on the RMB under UV irradiation. Therefore, this film material can be developed as a food packaging material with an anti-ultraviolet light function, slowing the oxidation rate of the contents.

### 3.4. Hydrophobicity and Morphology Analysis

**Figure 5:**
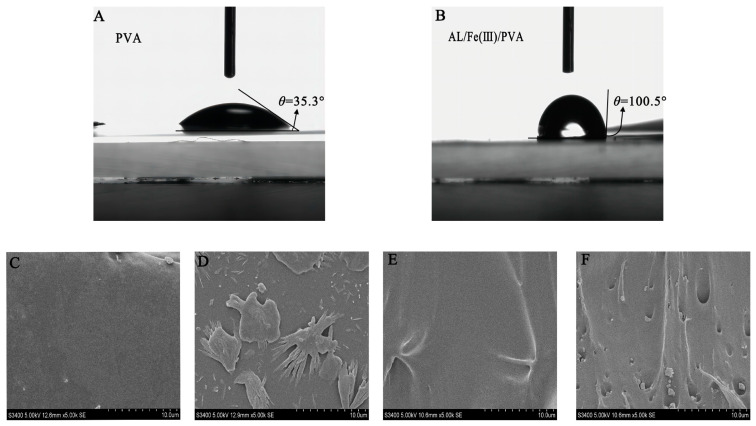
The water contact angles of the PVA film (**A**) and the AL/Fe(III)/PVA film (**B**). The microscopic surface morphology of the PVA film (**C**) and the AL/Fe (III)/PVA film (**D**). The cross-sectional morphology of the PVA film (**E**) and the AL/Fe (III)/PVA film (**F**).

Figure 5A,B show the water contact angles of different films. The results showed that the water contact angles of the PVA film and AL/Fe(III)/PVA film were 35.3° and 100.5°, respectively. PVA contains a large number of hydroxyl groups and is a hydrophilic material [35]. Therefore, according to the principle of surface chemistry, the water contact angle of the PVA film is small. Lignin is insoluble in water, and even though it was modified by ammonia, the hydrophobicity of the composite film was further improved by adding it to PVA. Therefore, the water contact angle of the composite film was larger than that of the PVA film. Additionally, the AL/Fe(III)/PVA film demonstrated a certain level of water resistance.

Figure 5C,D show the microscopic surface morphology of the PVA film and the AL/Fe(III)/PVA film. The results showed that the surface of the pure PVA film was smooth, and there were no large visible particles present even after being magnified 5000 times. The surface of the AL/Fe(III)/PVA film exhibited visible lignin particles and uneven concave–convex states. Figure 5E,F show the cross-sectional morphology of the PVA film and the AL/Fe (III)/PVA film. The results indicated that the cross-section of the pure PVA film exhibited a uniform and smooth state. When AL/Fe(III) was added to the mixture matrix, this resulted in the formation of a rough cross-section of the film. However, the mechanical analysis results showed that this small phase transition does not lead to the deterioration of the mechanical properties.

### 3.5. Slow-Release Performance of the AL/Fe(III)/PVA Film

**Figure 6:**
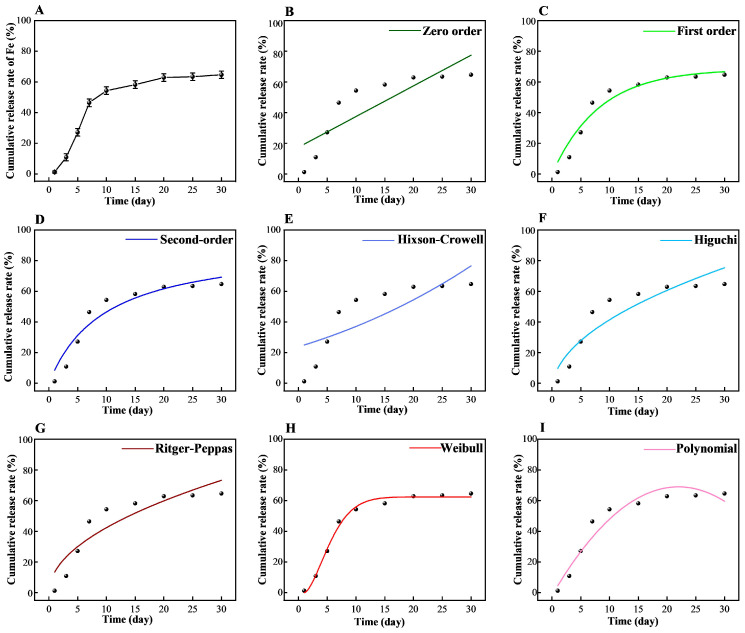
The release rate of iron of the AL/Fe(III)/PVA film in the soil (**A**). Kinetic models for the AL/Fe(III)/PVA film in the soil (**B**–**I**).

Figure 6A shows the cumulative release rate of the iron element from the AL/Fe(III)/PVA film into the soil. The release rate of iron displayed an S-shaped trend. Within 3 days, the release rate of iron was relatively slow. This was deemed to be due to the film material having a certain level of hydrophobicity, which can inhibit the dissolution of the iron elements to a certain extent. From 3 days to 10 days, respectively, the release rate of iron increased significantly, which was determined to be due to the water molecules entering the membrane material and dissolving the iron; meanwhile, the film material exhibited a certain degree of degradation, resulting in the rapid release of iron. The release rate leveled off between 10 days and 30 days, respectively, probably because the aminated lignin contained functional groups with some chelating ability, including the phenolic, hydroxyl, carboxyl, and ammonium groups, which formed a reticulation structure inside the film [39], thus slowing down the cumulative release rate of the iron element. If the nutrient release rate of the material is less than 15% and 75% in 1 day and 30 days, respectively, this material meets the requirements as a slow-release fertilizer [40,41]. In this study, the 1 day, 3 days, and 30 days release rates of iron from the AL/Fe(III)/PVA film reached 1.23%, 10.88%, and 64.67%, respectively. This suggests that the AL/Fe(III)/PVA film exhibits the slow-release effect of the iron element and can be used as a packaging material with a slow-release performance.

In addition, according to the experimental data of iron release from the composite film, the slow-release curves of iron were fitted using different dynamic models. The fitting results are shown in Table 1 and Figure 6. The determination coefficient R^2^ was used to evaluate the fitting degree. From the fitting equations and determination coefficient R^2^, the validity sequence of the fitted slow-release model was as follows: Weibull model > First-order kinetic model > Polynomial fitting model > Second-order dynamic model > Higuchi model > Ritger–Peppas model > Zero-level dynamics model > Hixson–Crowell model.

The Weibull model indicated that the release law of the iron elements in the film conformed to the dissolution law of solid drug formulations in sustained-release media [42]. The AL/Fe(III) complex was mixed in the PVA matrix as with small solid particles. When the film material was washed by water, the small particles of the AL/Fe(III) complex on the surface of the material entered the soil with the water flow, and the iron ions were released, resulting in a slow and uneven release of the iron element in the PVA matrix [43]. The results of the polynomial model fit showed that the second-order polynomial equation can better characterize the release kinetics of the iron nutrients and is convenient for practical calculation and application. The Higuchi model showed that these fertilizers were released through dissolution and diffusion [44]. When the film fertilizer was immersed in water, several complex iron ions were flushed into the soil by the water. At the same time, the polymer chains of PVA expanded. Since the film structure was highly cross-linked, van der Waals forces between the polymer chains further limited the migration of the polymer chains. As a result, the dissolution of the iron ions from the film was slowed down. The N value of the Ritger–Peppas model was in the range from 0.45 to 0.89, respectively, indicating that the drug release behavior observed was the result of a combination of drug diffusion and skeletal dissolution [45]. The fitting of the Ritger–Peppas model (n = 0.49818) showed that the release of iron from the film was regulated through the water diffusion and polymer relaxation mechanisms. Therefore, the iron release mechanism of the AL/Fe(III)/PVA film may be that the AL/Fe(III) complex on the surface of the film was dissolved first, and then the iron elements inside the material dissolved and diffused with the dissolution of the AL/Fe(III)/PVA matrix. Future work could consider a further analysis of the relationship between the degradation and release performance of these film materials in the soil.

## 4. Conclusions

In this study, a lignin-based film packaging material with an iron content of 1.06 wt% was prepared using the flow casting method. The AL/Fe(III) complex was prepared by impregnating the aminated lignin in the Fe(NO_3_)_3_ solution with a concentration of 5% by weight. The AL/Fe(III)/PVA film was prepared under the following conditions: the mass ratio of AL/Fe(III) to PVA was 3:7, the pH value of the reaction solution was 8, the reaction temperature was 85 °C, and the reaction time was 60 min. The water contact angle of the AL/Fe(III)/PVA film was 100.5°. Almost 100% of the UVB (325–275 nm) and UVC (275–200 nm) spectrum, as well as most of the UVA (400–325 nm) spectrum, was shielded by the film. The Weibull model was suitable for describing the release rate of the film in the soil. The film can be used for processing into planting bowls, transplanting pots, and food packaging material with an anti-ultraviolet light function. In addition, the molten AL/Fe(III)/PVA material can be used for root treatment during seedling transport. The wet soil located close to the seedling roots can be covered and fixed with the molten AL/Fe(III)/PVA material using the proper spraying processes. The slow release of the nutrient iron is beneficial in promoting chloroplast synthesis in seedlings, thus shortening the slow seedling stage.

## Figures and Tables

**Table 1:** Model fitting results of the release experiment.

Model	Fitting Equation	*R* ^2^
Zero-level dynamics model	*Q*_t_ = 2.00122*t* + 17.45839	0.81962
First-order kinetic model	*Q*_t_ = 68.46565 × (1 − 2.71828^−0.12195*t*^)	0.93705
Second-order dynamic model	*Q*_t_ = 9.43917*t* ÷ (1 + 0.10377*t*)	0.91550
Hixson–Crowell model	*Q*_t_ = (0.04559 + 2.87863)^3^	0.58108
Higuchi model	*Q*_t_ = 14.66114*t*^1/2^ − 4.96457	0.83582
Ritger–Peppas model	*Q*_t_ = 13.46392 × (*t*^0.49818^)	0.82712
Weibull model	*Q*_t_ = 62.46944 × (1 − $2.71828^{-{(0.18463\left(t\;-\;1)\right)}^{1.60988}}$)	0.98988
Polynomial fitting model	*Q*_t_ = 6.42995*t* − 0.14919*t*^2^ − 1.71071	0.92247

## Data Availability

Data are contained within the article.
